# P2Y_2_R Deficiency Attenuates Experimental Autoimmune Uveitis Development

**DOI:** 10.1371/journal.pone.0116518

**Published:** 2015-02-18

**Authors:** Lia Judice M. Relvas, Maya Makhoul, Remi Dewispelaere, Laure Caspers, Didier Communi, Jean-Marie Boeynaems, Bernard Robaye, Catherine Bruyns, François Willermain

**Affiliations:** 1 Dpt of Ophthalmology, CHU St-Pierre and Brugmann, Brussels, Belgium; 2 The Institute of Interdisciplinary Research, IRIBHM, Brussels, Belgium; 3 The Institute of Interdisciplinary Research, IRIBHM, Gosselies, Belgium; 4 Dpt of Laboratory Medicine, Erasme Hospital, Brussels, Belgium; 5 Université Libre de Bruxelles, Brussels, Belgium; Boston University School of Medicine, UNITED STATES

## Abstract

We aimed to study the role of the nucleotide receptor P2Y_2_R in the development of experimental autoimmune uveitis (EAU). EAU was induced in P2Y_2_^+/+^ and P2Y_2_^-/-^ mice by immunization with IRBP peptide or by adoptive transfer of in vitro restimulated semi-purified IRBP-specific enriched T lymphocytes from spleens and lymph nodes isolated from native C57Bl/6 or P2Y_2_^+/+^ and P2Y_2_^-/-^ immunized mice. Clinical and histological scores were used to grade disease severity. Splenocytes and lymph node cell phenotypes were analyzed using flow cytometry. Semi-purified lymphocytes and MACS-purified CD4^+^ T lymphocytes from P2Y_2_^+/+^ and P2Y_2_^-/-^ immunized mice were tested for proliferation and cytokine secretion. Our data show that clinical and histological scores were significantly decreased in IRBP-immunized P2Y_2_^-/-^ mice as in P2Y_2_^-/-^ mice adoptively transfered with enriched T lymphocytes from C57Bl/6 IRBP-immunized mice. In parallel, naïve C57Bl/6 mice adoptively transferred with T lymphocytes from P2Y_2_^-/-^ IRBP-immunized mice also showed significantly less disease. No differences in term of spleen and lymph node cell recruitment or phenotype appeared between P2Y_2_^-/-^ and P2Y_2_^+/+^ immunized mice. However, once restimulated in vitro with IRBP, P2Y_2_^-/-^ T cells proliferate less and secrete less cytokines than the P2Y_2_^+/+^ one. We further found that antigen-presenting cells of P2Y_2_^-/-^ immunized mice were responsible for this proliferation defect. Together our data show that P2Y_2_^-/-^ mice are less susceptible to mount an autoimmune response against IRBP. Those results are in accordance with the danger model, which makes a link between autoreactive lymphocyte activation, cell migration and the release of danger signals such as extracellular nucleotides.

## Introduction

Several factors are required to trigger an autoimmune organ-specific disease. Genetic and environmental components will collaborate to cause a breakdown of the peripheral immune tolerance (activation of autoreactive clones) and activation of resident cells of affected tissues [1]. Autoimmune uveitis (AIU) illustrates these autoimmunity paradigms. Genetic susceptibility of individuals with AIU has been widely studied and the association of several polymorphisms at different loci of the HLA system is well known [2]. Similarly, different groups have demonstrated the presence of autoreactive T cells and the role of cytokines released by these cells in the activation of resident cells of the eye during development of AIU [3].

Several data also attest the importance of the role of danger signals during these two key phases of pathological activation [4]. If a central place was given to exogenous danger signals, in particular microbial, the importance of endogenous danger signals began to emerge [5]. In this context, nucleotides are an important family of potential endogenous danger signals. In fact, normally, they are present almost exclusively within the cells. However, during cellular destruction or stress, like inflammation, nucleotides can be released in various amounts in the extracellular space and activate nucleotide receptors belonging to the P2X and P2Y families [6]. In many cases, the activation of P2 receptors results in an increase of the inflammatory process [7]. In agreement with the danger signal theory, we have shown that different nucleotides, ATPγS, UTP and UDP, are involved in the activation of retinal pigment epithelium and increase the basal as well as the TNFα-induced release of IL-8 [8]. Similarly, several works have demonstrated that nucleotides also profoundly influence antigen presentation and lymphocyte activation [9]. Accordingly, Granstein et al have shown, in vivo, that extracellular nucleotides strongly increase lymphocyte activation after systemic immunization [10].

In this work, we have thus hypothesized that nucleotides can act as danger signals during AIU and interfere with both the activation of autoreactive lymphocytes and the stimulation of blood retinal barrier cells. Using the native and adoptive transfer models of experimental autoimmune uveitis (EAU) we have compared the induction of uveitis in P2Y_2_R wild-type (P2Y_2_^+/+^) and KO mice (P2Y_2_^-/-^). In parallel, we have also compared in vitro the cell recruitment, phenotype, proliferation and cytokine secretion between P2Y_2_^+/+^ and P2Y_2_^-/-^ IRBP (interphotoreceptor retinoid binding protein)-immunized mice.

## Material and Methods

### Reagents and animals

IRBP peptide 1–20 (GPTHLFQPSLVLDMAKVLLD), representing residues 1–20 of human IRBP, was synthesized by New England Peptide (Gardner, MA USA). Pertussis toxin (PTX) and complete Freund’s adjuvant (CFA) were purchased from Sigma-Aldrich (Bornem, Belgium). Pathogen-free C57BL/6 male mice were purchased from Janvier (Genest St Isle, France). P2Y_2_R wild-type (P2Y_2_^+/+^) and KO (P2Y_2_^-/-^) mice on C57Bl/6 background were generated by one of the authors (B.R.) as previously described [11]. All mice were housed and maintained at the animal facilities in accordance with European guidelines. Animal treatment conformed to the ARVO Statement for the Use of Animals in Ophthalmic and Vision Research. Euthanasia of mice were conducted in CO2 chambers. This research was approved by the CEBEA (Commission d’Ethique du Bien-Etre Animal). The agrement number of the institute is LA: 1230332. All cells were cultured in RPMI 1640 medium supplemented with 25 mM HEPES, 10% fetal bovine serum, 1% L-glutamine, 1% sodium-pyruvate, 100 IU/ml penicillin, and 100g/ml streptomycin and 5.10^–5^M β-mercaptoethanol in a 5% CO2 and 95% humidity incubator.

### EAU induction

EAU was induced by injecting subcutaneously 100 μl of a mixture of 500 μg IRBP peptide 1–20 emulsified in CFA and supplemented with 2.5 mg/ml killed Mycobacterium Tuberculosis. All animals received at the same time an intraperitoneal injection of a single dose of 1.5 μg/100 μl PTX.

### Adoptive transfer model of EAU

EAU was also induced by adoptive transfer of autoreactive semi-purified IRBP 1–20 peptide-specific enriched T lymphocytes following a protocol adapted from Shao H et al [12]. Briefly, naïve C57BL/6 or P2Y_2_^+/+^ and P2Y_2_^-/-^ mice were immunized as for EAU induction. Twelve days later, mice were sacrificed and their spleen and draining lymph nodes collected. Splenic T cells were semi-purified by passage on nylon wool fiber columns, pooled with total lymph node cells and cultured with 10 μg/ml IRBP 1–20 peptide for 2 days before being injected i.p into naïve C57BL/6 or P2Y_2_^+/+^ and P2Y_2_^-/-^ mice (3x10^6^ cells/mouse).

### Clinical grading

A clinical grading was performed at days 7, 14 and 21 after EAU induction. For this purpose, animals were anesthetized by a 50 μl intramuscular injection in the leg of a Rompun (0.2%) and Ketalar (20 mg/ml) mixture. Eyes were dilated with tropicamid (5mg/ml) and phenylephrin (1.5 g/ml) and examined under the slit-lamp of a surgical microscope (Zeiss, Göttingen, Germany) by using a cover slip coated with a viscoelastic gel (Vidisic, Tramedico, Belgium) and positioned on the cornea. The clinical grading system used has been adapted from Xu H. et al. [13]. Briefly, vitritis, optic neuropathy, vasculitis and retinitis were separately scored in each eye, from 0 (no disease) to 4 (highly severe disease) with half points increments. The clinical score attributed to one mouse corresponds to the mean of the 4 parameters average of the 2 eyes.

### Histological grading

For histological grading, the mice were sacrificed 21 days after EAU induction. The eyes were collected, prefixed for 6 h at 4°C in PFA (paraformaldehyde) 4%, sucrose 3% and then put in three successive baths of 5%, 10% and 20% sucrose in PBS, respectively, for 24h each. Entire eyes were embedded in OCT (Sakura) and cut in 10 μm frozen sections using a cryostat (CM3050S Leica). A classical hematoxylin-eosin staining was then realized. The severity of EAU was evaluated on six sections, cut at different levels in each eye, and scored on a scale from 0 (no disease) to 4 (maximum disease) with half points increments, according to lesion type, size, and number by using the R. Caspi’s histological grading system [14]. In brief, the minimal criterion to score an eye as positive by histopathology is an inflammatory cell infiltration of the ciliary body, choroid, or retina (EAU grade 0.5). Progressively higher grades are assigned for the presence of discrete lesions in the tissue, such as vasculitis, granuloma formation, retinal folding or detachment, and photoreceptor damage. The histological score attributed to one mouse corresponds to the mean of the inflammatory lesions average of the 2 eyes.

### In vitro characterization of cell recruitment, phenotype, proliferation and cytokine secretion

Twelve days after immunization with IRBP 1–20 peptide, mice were sacrificed and their spleen and draining lymph nodes collected. Splenic T lymphocytes were semi-purified by passage on nylon wool fiber columns and total lymph node (LN) cells recovered by cell dissociation. Each cell type was characterized by flow cytometry for cellular phenotype by using FITC- or PE- anti-mouse antibodies directed against CD3, CD11b, CD11c, MHCII molecules, OX40, OX40L and CCR6 (BD Pharmingen).

Semi-purified splenic T cells and LN cells were also pooled and recultured in vitro, for 48h, with IRBP 1–20 peptide before being tested for cell proliferation and cytokine secretion. For cell proliferation, 5.10^5^ cells/200 μl/well were seeded in 96-wells plates, in complete RPMI medium alone or supplemented with IRBP 1–20 peptide (10 ng/ml) or with Dynabeads Mouse T-activator CD3/CD28 (5μl/well, Invitrogen). T cell proliferation was measured on day 3, by thymidine incorporation, after an 18 hours pulse with 1 μCi/well (^3^H) thymidine (Perkin Elmer, Zaventem, Belgium). To measure cytokine production 10^6^ cells/well/1 ml were seeded in 24-wells plates, in complete RPMI medium alone or supplemented with IRBP 1–20 peptide (10 μg/ml). Supernatants were collected after 48h and cytokine secretion quantified by specific ELISA, following manufacturer’s instructions (IFNγ and TNFα: Biosource; IL-17α: R&D Systems). Within each experiment, cell proliferation and cytokine secretion were measured after pooling cells from 4 animals in each group. Due to inter-experiment variations in absolute values, repeat experiments could not be combined. Patterns of response were however highly reproducible. Figures depict representative experiments.

### Intracytoplasmic cytokine secretion

Same semi-purified splenic T cells pooled with LN cells were also processed for a CD4+ T cell-dependent detection of intracytoplasmic secretion of IFNγ and IL-17. Briefly, after the 48h-culture in presence of IRBP 1–20 peptide (10 μg/ml), the cells were first restimulated for 5 h with PMA/ionomycin (at 50 ng/ml and 1 μg/ml respectively, Sigma-Aldrich, Bornem, Belgium) in the presence of GolgiStop (1 μl/ml, BD Biosciences), a protein transport inhibitor. Cells were then washed, fixed and permeabilized (Cytofix/Cytoperm, BD Biosciences) before being incubated with the Mouse Th1/Th17 Phenotyping Cocktail (BD Biosciences) following manufacturer’s instructions. Samples were analyzed by flow cytometry for intracytoplasmic IFN-γ and IL-17 production by CD4+ T cells.

### Real-time PCR

Twelve days after immunization with IRBP 1–20 peptide, mice (n = 5/group) were sacrificed and their spleen collected and dissociated. Splenic cells were proceeded for qRT-PCR analysis of CD_3_ and Foxp3 gene expression. β-Actin was used as a non-modulated reference gene. The mRNA extraction and isolation were carried out using the automated MagNA Pure LC Instrument system and the MagNAPure LC mRNA Isolation Kit II, following manufacturer’s instructions (Roche Applied Science, Vilvoorde, Belgium). A one step real-time quantitative PCR technique using the RNA Master Hybridization Probes Kit (Roche Applied Science) was used to quantify mRNA expression using specific primes and fluorescent probes for mouse CD_3_, Foxp3 and β-actin (Applied Biosystems). Data are presented as relative expression of Foxp3 versus CD_3_.

### Mixed lymphocyte reaction (MLR)

MLR were performed between T lymphocytes and antigen-presenting cells (APC) of P2Y_2_^+/+^ and P2Y_2_^-/-^ IRBP-immunized mice in autologous and cross-culture conditions in order to assess their responder and activation capacities, respectively. Briefly, CD4+ T cells were isolated from the draining lymph nodes of day-12 IRBP-immunized P2Y_2_^+/+^ and P2Y_2_^-/-^ mice by using the CD4+ T cell isolation Kit II and a MACS negative selection, according to the manufacturer’s protocol (Miltenyi Biotec). The CD4+ T cell purity was confirmed by flow cytometry and was over 94%. Irradiated (30 Gy) splenocytes isolated from same mice were used as stimulator APC. T lymphocytes (2.10^5^ cells/100 μl/well) and APC (10^6^ cells/100 μl/well) were co-cultured in 96-well plates in complete medium alone or supplemented with IRBP 1–20 peptide (10 ng/ml). T-cell proliferation was measured on day 3, by thymidine incorporation, after an 18 hours pulse with 1 μCi/well (^3^H) thymidine (Perkin Elmer).

### Statistics

EAU clinical and histological scores were expressed as medians and compared using the Mann-Whitney test. Data from cell proliferation and cytokine secretion were statistically analyzed with the unpaired *t* test, using a Welch correction if needed.

## Results

### P2Y_2_ receptor deficiency attenuates EAU development

**Fig 1:**
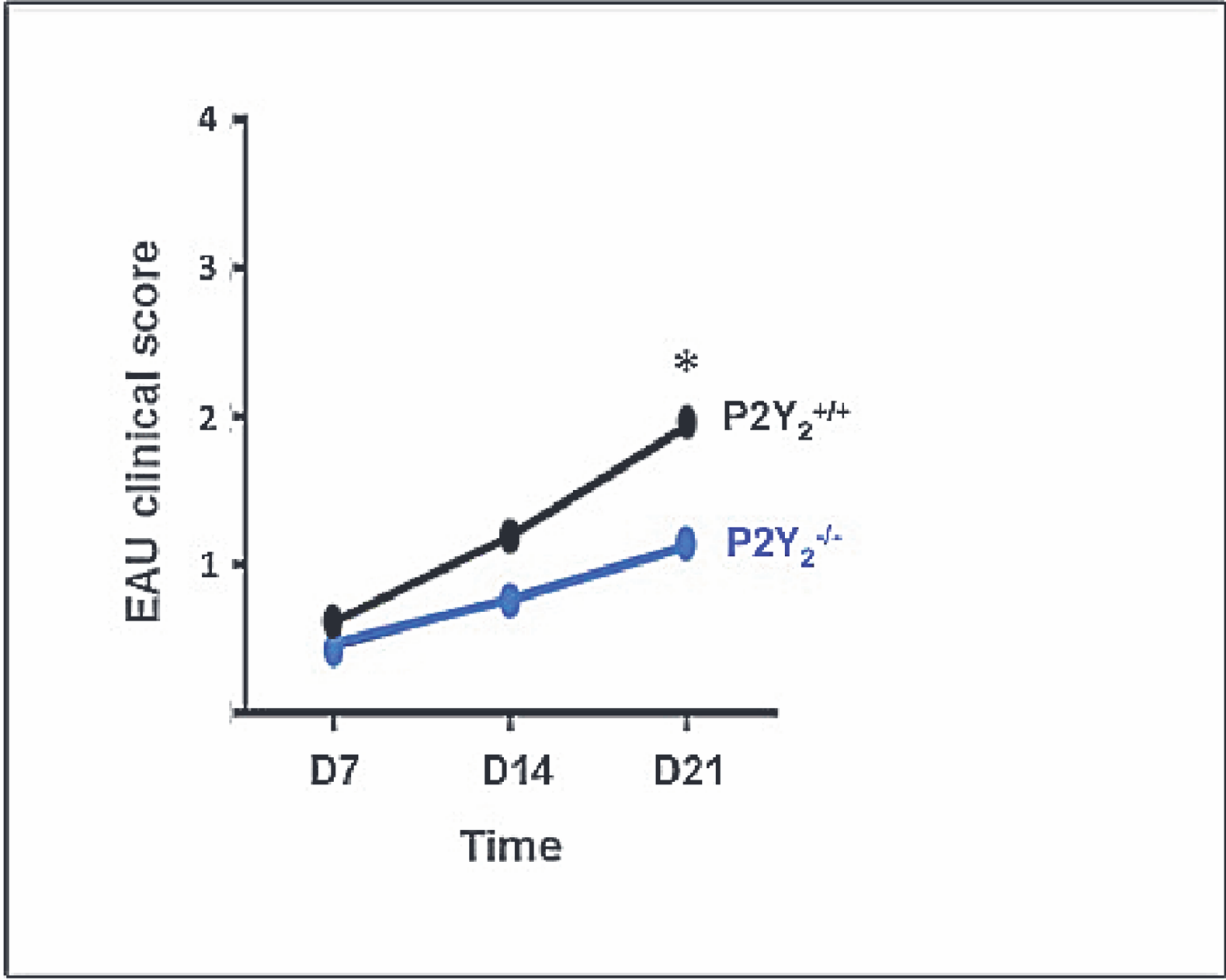
P2Y_2_^-/-^ mice are partially protected against uveitis development. EAU was induced in P2Y_2_^+/+^ (n = 15 ->12) and P2Y_2_^-/-^ (n = 15 ->10) mice by s.c. immunization with IRBP 1–20 peptide emulsified in CFA and i.p. injection of PTX. A blind fundoscopy was performed at days 7, 14 and 21 to detect vitritis, optic neuropathy, vasculitis and retinitis. Data represent the median of EAU clinical scores established for all mice at each time point, in the two groups. Three independent experiments were done, starting with 5 animals in each group. *p < 0,05.

To investigate the possible role of P2Y_2_ receptors (P2Y_2_R) in the development of intraocular inflammation, we first immunized P2Y_2_^+/+^ and P2Y_2_^-/-^ mice with IRBP 1–20 peptide plus CFA and PTX. We next performed a fundus examination by a masked observer at days 7, 14 and 21 to monitor disease severity. A clinical score was then established for each eye of every animal in the two groups. Fig. 1 shows that P2Y_2_^-/-^ mice are less severally affected with a statistically significant difference at day 21.

### P2Y2 receptor deficiency attenuates EAU development induced by the adoptive transfer of semi-purified IRBP-specific TL (efferent phase study)

**Fig 2:**
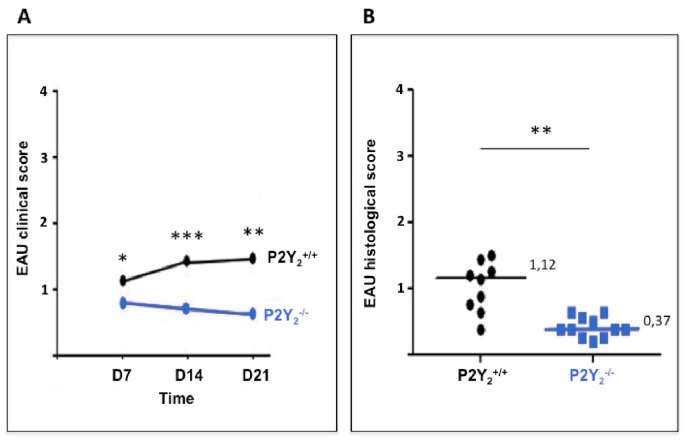
P2Y_2_^-/-^ mice are partially protected against uveitis development induced by the adoptive transfer of IRBP-specific enriched TL—Efferent phase. P2Y_2_^+/+^ (n = 9) and P2Y_2_^-/-^ (n = 11) mice have received an adoptive transfer of semi-purified IRBP-specific enriched TL from C57Bl/6 immunized mice. Data are from two independent experiments. (A) A blind fundoscopy was performed at days 7, 14 and 21 and an EAU clinical score was established for each eye of every animal in the two groups. Data represent the median of clinical scores established for all mice at each time point. (B) At day 21, all the mice were sacrificed, the eyes enucleated and processed for histology. Hematoxylin-eosine coloration was realized in order to perform a double blind histological grading. Each symbol represents the established EAU histological score for one mouse. Lines represent median histological scores. *P < 0,05; **P < 0,01; ***p < 0,001.

We further investigated the effect of P2Y_2_R deficiency on EAU development by performing an adoptive transfer of semi-purified IRBP 1–20 peptide-specific enriched T lymphocytes (TL) isolated from a group of IRBP-immunized C57Bl/6 mice into P2Y_2_^+/+^ and P2Y_2_^-/-^ mice. A masked observer performed a fundus examination, at days 7, 14 and 21 to monitor the severity of the disease. A clinical score was then established for each eye of every animal in the two groups. As revealed on Fig. 2A, P2Y_2_^-/-^ mice developed, at each time point, a significant less severe disease after TL adoptive transfer, as compared to P2Y_2_^+/+^ mice. At day 21, after the last fundus examination, all animals were sacrificed, eyes prepared for histological analysis and a histological grading performed by two masked observers. As shown on Fig. 2B, the histological score obtained at day 21 was also significantly different, the eyes from P2Y_2_^-/-^ mice being less severely affected than those from P2Y_2_^+/+^ mice.

### P2Y_2_ deficiency decreases the capability of semi-purified IRBP-specific enriched TL to induce uveitis (afferent phase study)

**Fig 3:**
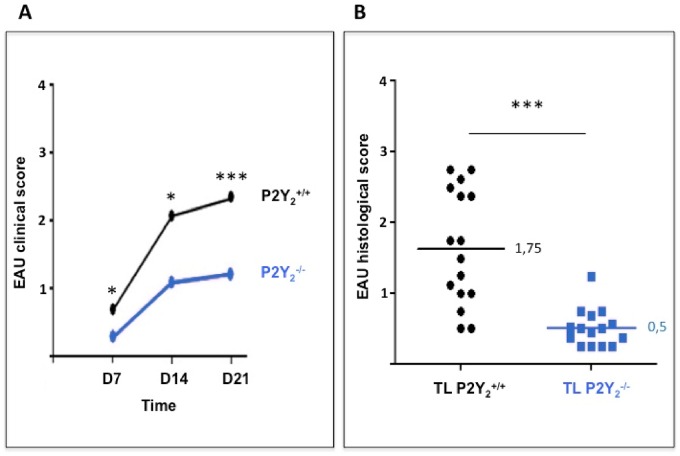
In vivo decreased capacity of IRBP-specific enriched TL from P2Y_2_^-/-^ mice to induce uveitis—Afferent phase. Semi-purified IRBP-specific enriched TL were recovered from five P2Y_2_^+/+^ and P2Y_2_^-/-^ immunized mice, pooled and adoptively transferred in two groups of naïve C57Bl/6 mice after a 48h in vitro restimulation with IRBP. Data are from three independent experiments where 16 mice in all received P2Y_2_^+/+^ TL and 15 mice P2Y_2_^-/-^ TL. (A) A blind fundoscopy was performed at days 7, 14 and 21 and a clinical score was established for each eye of every animal in the two groups. Data represent the median of clinical scores established for all mice at each time point. (B) At day 21, all the mice were sacrificed, the eyes enucleated and processed for histology. Hematoxylin-eosine coloration was realized in order to performed a double blind histological grading. Each symbol represents the established histological score for one mouse. Lines represent median histological scores. * *p* < 0,05; ***p < 0,001.

We have next analyzed the role of P2Y_2_R expression on the activation of autoreactive lymphocytes, by performing an adoptive transfer of semi-purified IRBP-specific enriched TL from P2Y_2_^-/-^ or P2Y_2_^+/+^ immunized mice into naïve C57Bl/6 mice. A masked observer performed a fundus examination, at days 7, 14 and 21 to monitor the severity of the disease. A clinical score was then established for each eye of every animal in the two groups. As shown in Fig. 3A, the induced disease was significantly less severe in mice having received an adoptive transfer of TL from P2Y2^-/-^ immunized mice. At day 21, after the last fundus examination, all animals were sacrificed, eyes prepared for histological analysis, and a histological grading performed by two masked observers. Data from Fig. 3B show that the histological score is also lower in the eyes of mice having received semi-purified IRBP-specific enriched TL from P2Y_2_^-/-^ immunized animals.

We have further purified the autoreactive CD4+ TL from these P2Y_2_^-/-^ or P2Y_2_^+/+^ immunized mice and have tested their capability to induce uveitis by adoptive transfer into naïve C57Bl/6 mice. Our results showed similarly decreased efficacy of purified TL from P2Y_2_^-/-^ immunized mice in inducing EAU as compared to the P2Y_2_^+/+^ counterparts since the median of clinical scores were 0.625 versus 1.375, respectively, with a significant p value of 0.034.

### P2Y_2_ deficiency does not affect spleen and lymph node TL/APC balance after IRBP 1–20 peptide immunization

**Fig 4:**
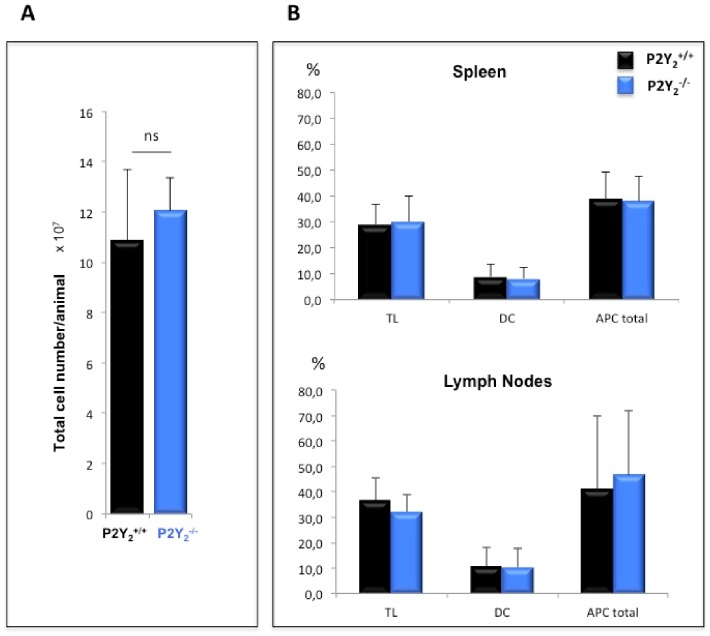
No difference in cell number or in cell phenotype between P2Y_2_^+/+^ and P2Y_2_^-/-^ IRBP-immunized mice. Twelve days after s.c. immunization with IRBP 1–20 peptide emulsified in CFA and combined with an i.p. injection of PTX, P2Y_2_^+/+^ and P2Y_2_^-/-^ mice were sacrificed and their spleen and draining lymph nodes collected and dissociated. (A) Total cell number count of mixture of splenic T lymphocytes semi-purified by passage on nylon wool fiber columns and LN cells. Mean +/- SEM. (B) Spleens and LN were independently characterized by flow cytometry for cellular phenotype by using FITC- or PE- anti-mouse antibodies directed against CD3, CD11b, CD11c and MHCII surface molecules. TL: CD3+; DC: CD11c+/CD11b+ or MHCII+; APC tot: DC + CD11c-/CD11b+ or MHCII+. Mean +/- SEM. Data are from at least 10 different mice in each group.

In order to investigate the mechanisms responsible for the decreased susceptibility of P2Y_2_^-/-^ mice or TL to develop or induce uveitis, respectively, we have first analyzed comparatively, 12 days after immunization with IRBP 1–20 peptide in CFA and Mycobacterium plus PTX, the spleen and draining lymph nodes of P2Y_2_^-/-^ and P2Y_2_^+/+^ IRBP-immunized mice for cell composition. As shown in Fig. 4, we did not find differences between P2Y_2_^-/-^ and P2Y_2_^+/+^ immunized mice in total cell count (Fig. 4A) or in the expression of CD3, CD11c, CD11b and MHCII surface molecules (Fig. 4B). Same proportions of TL, DC and total APC (DC + macrophages) were thus induced in both P2Y_2_^-/-^ and P2Y_2_^+/+^ mice by IRBP 1–20 peptide immunization.

### Semi-purified lymphocytes from P2Y_2_^-/-^ immunized mice proliferate less and secrete less cytokines in response to IRBP 1–20 peptide in vitro restimulation

**Fig 5:**
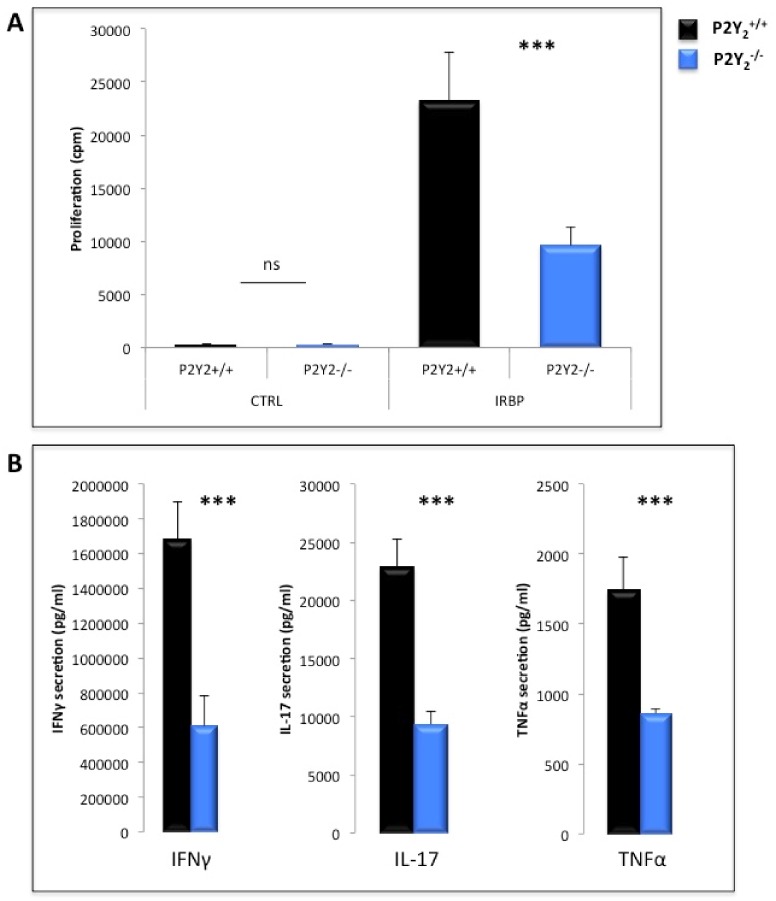
P2Y_2_^-/-^ TL proliferate less and secrete less cytokines in response to in vitro IRBP restimulation. Twelve days after s.c. immunization with IRBP 1–20 peptide, P2Y_2_^+/+^ and P2Y_2_^-/-^ mice were sacrificed and their spleen and draining lymph nodes collected and dissociated. (A) Splenic TL were semi-purified by passage on nylon wool fiber columns and pooled with LN cells before being in vitro restimulated in medium alone (CTRL) or supplemented with IRBP 1–20 peptide. TL proliferation was tested after 72h by (^3^H)- incorporation. Data are from a representative experiment of 10 with 4 mice per group. (B) Cytokine secretion was quantified, by specific ELISA, in culture supernatants after 48h of IRBP restimulation. Data are from a representative experiment of 5 or 6 or 3 respectively, with 4 mice per group. ns: not significant; **p < 0,01; ***p < 0,001.

Since the effect of P2Y_2_ deficiency could not be explained by a difference in secondary lymphoid organ recruitment of immunocompetent cells, we hypothesized that the absence of P2Y_2_R could functionally impact either the TL proliferation and cytokine secretion or the antigen presenting cell capabilities. Semi-purified TL from P2Y_2_^-/-^ and P2Y_2_^+/+^ immunized mice were thus cultured in vitro for 48h either in medium alone or supplemented with the IRBP 1–20 peptide and their proliferation measured by thymidine incorporation. Fig. 5A clearly shows that, in response to IRBP1–20 peptide restimulation, the TL from P2Y_2_^-/-^ immunized mice proliferate less than the TL from P2Y_2_^+/+^ mice. Moreover, as shown in Fig. 5B, they also secrete less IFN-γ, IL-17 and TNF-α. Using intracellular flow cytometry, further experiments were done in order to assess more precisely the CD4+ T cell-dependent secretion of IFN-γ and IL-17. Our results showed that, in response to IRBP1–20 peptide restimulation, 3.6% of CD4+ TL from P2Y_2_^+/+^ mice versus only 1.8% of CD4+ TL from P2Y_2_^-/-^ mice did secrete IFN-γ (p = 0.05). For the detection of CD4+ TL secreting IL-17, the assays were however not conclusive as the cell cultures were not polarized to amplify the Th17 subset.

### P2Y_2_ deficiency affects antigen presenting cell capabilities

**Fig 6:**
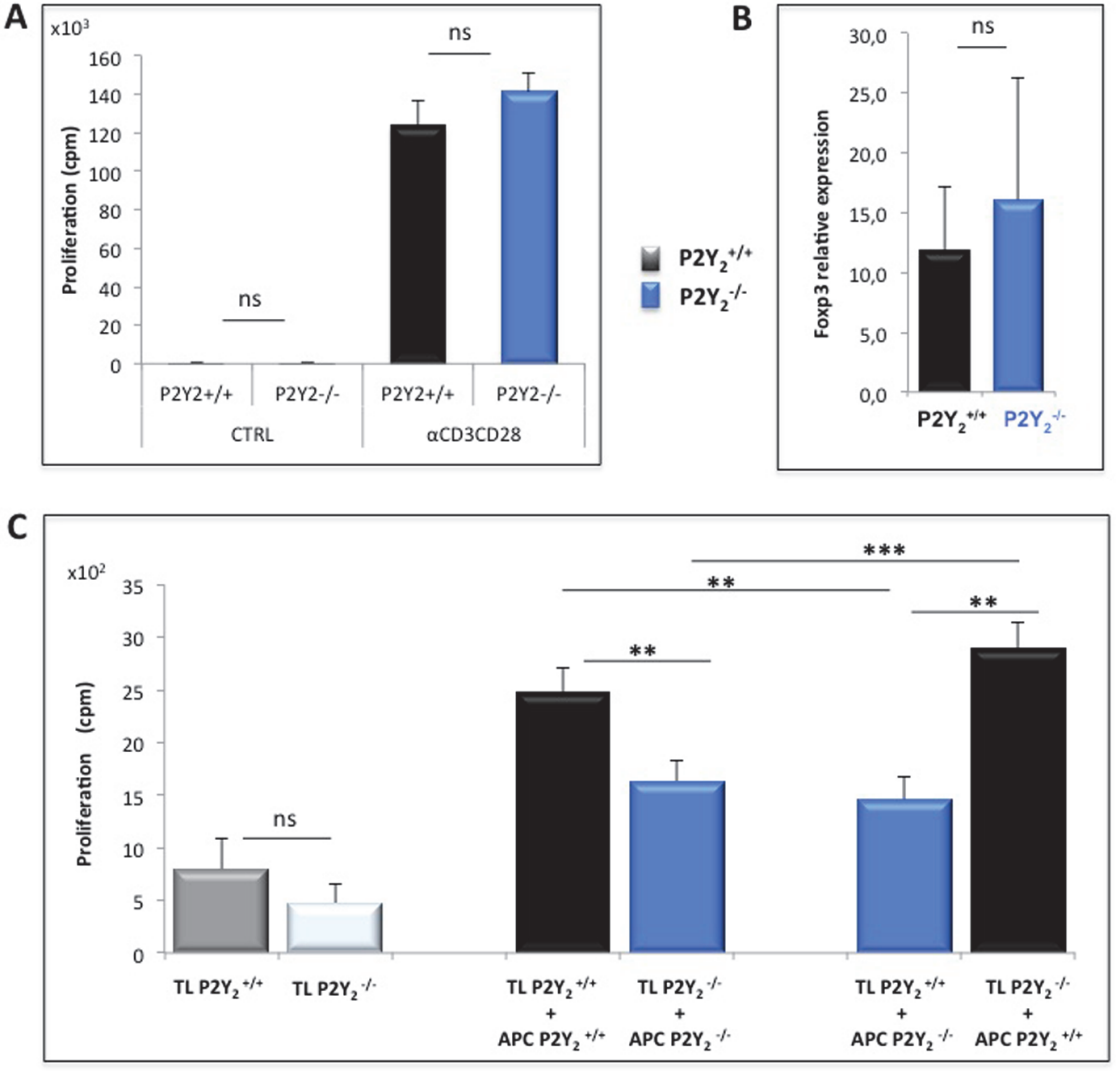
P2Y_2_^-/-^ reduced TL proliferation in response to in vitro IRBP restimulation is linked to a defect in P2Y_2_^-/-^ APC activation capacities. Twelve days after s.c. immunization with IRBP 1–20 peptide, P2Y_2_^+/+^ and P2Y_2_^-/-^ mice were sacrificed and their spleen and draining lymph nodes collected and dissociated. (A) Splenic TL were semi-purified by passage on nylon wool fiber columns and pooled with LN cells before being in vitro restimulated in medium alone (CTRL) or supplemented with Dynabeads Mouse T-activator CD3/CD28. TL proliferation was tested after 72h by (^3^H)-thymidine incorporation. Data are from a representative experiment of 6 with 4 mice per group. ns: not significant (B) Spleens from P2Y_2_^+/+^ and P2Y_2_^-/-^ IRBP-immunized were independently processed and analyzed by qPCR for β-Actin, CD3 and Foxp3 mRNA expression. Data are from 5 different mice in each group and are expressed as relative expression of Foxp3 versus CD3. ns: not significant (C) CD4+ TL were isolated from LN of P2Y_2_^+/+^ and P2Y_2_^-/-^ mice by magnetic separation and co-cultured with total irradiated (30 Gy) splenocytes (APC) in autologous and cross-culture conditions, in presence of IRBP 1–20 peptide. TL proliferation was tested after 72h by (^3^H)-thymidine incorporation. Data, expressed in cpm, are from a representative experiment of 3 with 4 mice per group. ns: not significant; **p < 0,01; ***p < 0,001.

As shown in Fig. 6A, the decreased proliferation of the P2Y_2_^-/-^ TL in response to IRBP1–20 peptide restimulation was not related to a default in TCR signaling pathway. Indeed, when semi-purified IRBP-specific enriched TL isolated from P2Y_2_^+/+^ or P2Y_2_^-/-^ immunized mice were restimulated in vitro with anti-CD3/CD28 coated beads they showed equal proliferation in response to TCR engagement. On the other hand, we have also investigated by qPCR the expression of Foxp3 mRNA in spleens of P2Y_2_^+/+^ and P2Y_2_^-/-^ IRBP-immunized mice and did not find a different proportion of Foxp3+ Treg among CD3+ TL between P2Y_2_^+/+^ and P2Y_2_^-/-^ immunized mice (Fig. 6B ).

The next step was then to analyze if P2Y_2_R deficiency could affect the capabilities of the antigen-presenting cells. For that purpose, P2Y_2_^-/-^ and P2Y_2_^+/+^ mice were immunized with IRBP 1–20 peptide in CFA and Mycobacterium plus PTX. 12 days after immunization, the lymph nodes were recovered and the CD4+ TL purified by depletion of magnetically labeled non-target cells. Those CD4+ lymphocytes from P2Y_2_^+/+^ or P2Y_2_^-/-^ immunized mice were co-cultured with irradiated splenocytes (used as APC) from the same P2Y_2_^+/+^ or P2Y_2_^-/-^ immunized mice, in medium alone or supplemented with IRBP 1–20 peptide. Fig. 6C illustrates proliferation data from all the different combinations of responder TL versus stimulator APC that have been tested in co-cultures in presence of IRBP 1–20 peptide. Results show that by reconstituting autologous culture conditions with TL and APC from either P2Y_2_^+/+^ or P2Y_2_^-/-^ immunized mice, we could observe similar reduced P2Y_2_^-/-^ TL proliferation as illustrated in Fig. 5A. Moreover, when P2Y_2_^+/+^ CD4+ TL were put in culture with P2Y_2_^-/-^ irradiated splenocytes, their proliferation was significantly decreased as compared to autologous P2Y_2_^+/+^ culture condition. On the opposite, when P2Y_2_^-/-^ CD4+ TL were put in culture with P2Y_2_^+/+^ irradiated splenocytes, their proliferation was restored to the one of P2Y_2_^+/+^ CD4+ TL. Altogether those data suggest that P2Y_2_R deficiency affects the antigen presenting cell capability of splenocytes. Moreover, they argue against a role of Treg in the observed effect of P2Y_2_R deficiency.

## Discussion

The development of autoimmune uveitis (AI) requires both the activation of retinal specific autoreactive lymphocyte clones, their migration to the eye and the breakdown of the blood retinal barrier (BRB) [1]. In this work, we found that P2Y_2_ deficiency attenuates EAU development and strongly affects the activation of IRBP-specific autoreactive lymphocytes after systemic immunization.

Our results are in accordance with the danger model, which makes a link between autoreactive lymphocyte activation, immune cell migration and the release of endogenous danger signals (DAMPs, damage associated molecular patterns) such as extracellular nucleotides. Among them, extracellular ATP (eATP) was recognized as an important modulator of immune responses through its binding to plasma membrane P2 purinergic receptors which are expressed by a wide range of cells [15]. In physiologic conditions, the concentration of eATP is quite low but it can be rapidly released in various amounts after cell stress, damage or death [16]. Then, eATP exerts immunostimulatory or immunosuppressive effects depending on its extracellular concentration (high or low, respectively), on which P2 receptors are engaged on specific immune cells and on the extent of the stimulation [15]. Roughly, murine models of inflammatory and autoimmune diseases have shown that eATP can act as a proinflammatory molecule not only by stimulating innate immune responses but also by favoring effector T-cell activation, mainly through P2X_7_ signaling. In our work, by using a specific gene knockout approach, we have focused on the effect of eATP on P2Y_2_R instead of using receptor specific antagonists. However, it is likely that other P2 receptors, including P2X receptors might also play a role in EAU development. In this context, two studies reported contradictory effect of P2X7 deficiency on experimental autoimmune encephalomyelitis development, either protective [17] or deleterious [18]. Yet, It has been shown also that the nucleotide (specially ATP) affinity for P2Y_2_ receptors is significantly higher than for P2X_7_ receptors (EC_50_ 0.1 μM vs 100 μM) [19]

In humans, several in vitro studies have pointed out a more complex role of eATP, able also to inhibit the cytokine secretion, proliferation or cytotoxic activity of immune cells such as DC, macrophages, NK cells and T lymphocytes through the activation of P2Y_11_ receptors [16]. Those P2Y_11_ receptors are however not expressed on murine cells. Another major fact to point out is that nucleotides are unstable short-lived molecules acting in an autocrine or paracrine manner. Especially, eATP is hydrolyzed into ADP/AMP and adenosine by plasma membrane-bound ectoenzymes, i.e. CD39 and CD73, whose expression on different immune cells or even on a same cell subset but in different location is quite variable. Therefore, a lot of studies evaluating the effect of eATP were done with ATPγS, a non-hydrolysable ATP analogue, rendering those data not (easily) comparable to ours.

As concerns our experimental model of EAU, we first induced a uveitis in P2Y_2_^+/+^ and P2Y_2_^-/-^ mice and observed reduced clinical scores in P2Y_2_^-/-^ animals. To our knowledge, there is no other publication on the role of the P2Y_2_ deficiency during the development of an experimental autoimmune disease.

We next demonstrated that P2Y_2_^-/-^ mice developed a significantly less severe disease after the adoptive transfer of semi-purified IRBP 1–20 peptide-specific enriched T lymphocytes (TL) isolated from C57Bl/6 immunized mice, as compared to P2Y_2_^+/+^ mice (efferent phase study). In order to evaluate a potential role of P2Y_2_R deficiency in lymphocyte migration, we similarly transferred Indium-radiolabeled TL and followed their in vivo migration with a SPECT camera. Unfortunately, this methodology appeared not sensitive enough to detect the presence of a small number of autoreactive TL in the eye. We however observed, in the few animals we have experienced, that the TL migration from the peritoneal cavity to peripheral organs was more important in P2Y_2_^+/+^ than in P2Y_2_^-/-^ mice (Lia J. M. Relvas et al, unpublished data). Besides, our findings are in line with several studies showing the importance of P2Y_2_R expression on epithelial and endothelial cells for VCAM1 expression and secondary recruitment of inflammatory cells [11,20,21].

Our data also showed that naïve C57Bl/6 mice, which have received an adoptive transfer of enriched TL from P2Y2-/- IRBP-immunized mice, displayed significantly lower disease (afferent phase study). Yet, no differences in term of spleen and lymph node cell recruitment or phenotype appeared between P2Y2-/- and P2Y2+/+ immunized mice. Nevertheless, once restimulated in vitro with IRBP, P2Y2-/- semi-purified T cells proliferate less and secrete less cytokines (IFNγ, IL-17 and TNFα) than the P2Y2+/+ one. The decreased proliferation of the P2Y2-/- TL in response to IRBP 1–20 peptide restimulation was not related to a default in TCR signaling pathway. Moreover, we did not observed a different proportion of Foxp3+ Treg among CD3+ TL between P2Y2+/+ and P2Y2-/- immunized mice. Together, our data on TL proliferation and Foxp3 expression strongly argue against a role of Treg in the observed effect of P2Y2R deficiency.

Lastly, our data showed that P2Y_2_R deficiency negatively affected more precisely the stimulatory capacities of the antigen-presenting cells and subsequently the lymphocyte activation. By addressing the potential mechanisms explaining the APC defects, we have detected within the IRBP-immunized P2Y_2_^-/-^ mice a trends toward a decreased expression of OX-40L, CCR6 and CXCL9, different molecules implicated in lymphocytic activation and migration [22–24] (Lia Judice Relvas, personnal communication). These results contrast with the findings of Müller T et al who described no significant differences in the priming capabilities of dendritic cells from P2Y_2_^-/-^ mice [25]. This discrepancy can be explained by important differences in experimental design.

Altogether our results are in agreement with the present literature showing the DAMPs properties of nucleotides. Hence, Idzko M et al have shown that extracellular ATP triggers and maintains asthmatic airway Th2 inflammation [26] and Granstein RD et al have demonstrated that ATPγS enhances cutaneous Th1 immune response [10]. However, our data showed that the DC migration toward secondary lymphoid organs was not influenced by P2Y_2_R deficiency. This somewhat contrasts with the description by Müller T et al or Communi D et al that P2Y_2_ receptors mediate DC lung chemotaxis during allergic inflammation[25] or pneumonia virus infection [27], respectively. Again, this discrepancy can be explained by profound differences in experimental protocols. First, we use two adjuvants for the induction of EAU: in addition to an i.p. injection of PTX inducing DC maturation [28] and identified as crucial for the emergence of the autoimmune pathology, the IRBP 1–20 peptide is indeed injected s.c. as emulsified in CFA enriched in heat-inactivated mycobacterium tuberculosis. Increased extracellular ATP concentrations could probably amplify adjuvant-mediated TLR stimulation of innate immune cells. The innate immune activation must thus be largely different in our model as compared to both lung models. Second, in a cutaneous immunization model, ATPγTP emulsified in CFA envating/maturing the resident antigen-presenting Langerhans cells [10], which is quite different from the recruitment of immature DC in bronchoalveolar fluids. Third, an exogenous ATPγS stimulation was performed and required in the lung model to observe the difference of dendritic cell migration, highlighting the unstable nature of the ATP released locally [26]. It is well-known that ATP exerts immunostimulant effects on DC/Langerhans cells [10]. Some studies argued for the implication of P2X_7_ receptors [29]; others suggested the enrolment of other P2 receptors [30]. Our work highlights such a role for P2Y_2_ receptors.

In conclusion, our data show that P2Y_2_^-/-^ mice are less susceptible to mount an autoimmune response against IRBP peptide 1–20, influencing the development of EAU. Those results are in accordance with the danger model, which makes a link between autoreactive lymphocyte activation, immune cell migration and the release of danger signals such as extracellular nucleotides. But, as compared to the literature, our results demonstrate for the first time an extension of the role of P2Y_2_R to TH1- and TH17-mediated autoimmune responses.
